# A Novel m^6^A Gene Signature Associated With Regulatory Immune Function for Prognosis Prediction in Clear-Cell Renal Cell Carcinoma

**DOI:** 10.3389/fcell.2020.616972

**Published:** 2021-01-21

**Authors:** Siteng Chen, Ning Zhang, Encheng Zhang, Tao Wang, Liren Jiang, Xiang Wang, Junhua Zheng

**Affiliations:** ^1^Department of Urology, Shanghai General Hospital, Shanghai Jiao Tong University School of Medicine, Shanghai, China; ^2^Department of Urology, Ruijin Hospital, Shanghai Jiao Tong University School of Medicine, Shanghai, China; ^3^Department of Pathology, Shanghai General Hospital, Shanghai Jiao Tong University School of Medicine, Shanghai, China

**Keywords:** N^6^-methyladenosine (m^6^A), clear cell renal cell carcinoma, WGCNA, regulatory immune function, nomogram

## Abstract

The important role of N^6^-methyladenosine (m^6^A) RNA methylation regulator in carcinogenesis and progression of clear-cell renal cell carcinoma (ccRCC) is poorly understood by now. In this study, we performed comprehensive analyses of m^6^A RNA methylation regulators in 975 ccRCC samples and 332 adjacent normal tissues and identified ccRCC-related m^6^A regulators. Moreover, the m^6^A diagnostic score based on ccRCC-related m^6^A regulators could accurately distinguish ccRCC from normal tissue in the Meta-cohort, which was further validated in the independent GSE-cohort and The Cancer Genome Atlas-cohort, with an area under the curve of 0.924, 0.867, and 0.795, respectively. Effective survival prediction of ccRCC by m^6^A risk score was also identified in the Cancer Genome Atlas training cohort and verified in the testing cohort and the independent GSE22541 cohort, with hazard ratio values of 3.474, 1.679, and 2.101 in the survival prognosis, respectively. The m^6^A risk score was identified as a risk factor of overall survival in ccRCC patients by the univariate Cox regression analysis, which was further verified in both the training cohort and the independent validation cohort. The integrated nomogram combining m^6^A risk score and predictable clinicopathologic factors could accurately predict the survival status of the ccRCC patients, with an area under the curve values of 85.2, 82.4, and 78.3% for the overall survival prediction in 1-, 3- and 5-year, respectively. Weighted gene co-expression network analysis with functional enrichment analysis indicated that m^6^A RNA methylation might affect clinical prognosis through regulating immune functions in patients with ccRCC.

## Introduction

It is estimated that there will be 73,750 new cases of renal malignant tumors in the United States in 2020 (Siegel et al., 2020). As one of the most aggressive malignancies, renal cell carcinoma (RCC) accounts for 2–3% of the malignancies (Ljungberg et al., 2015). Clinically, clear-cell RCC (ccRCC) is the most common type of renal carcinoma, representing ~80% of RCC. Although the therapy methods of ccRCC, including the surgical and targeted therapy, have been improved, the poor survival prognosis is also noticed in ccRCC, which is obligated to most metastatic cases of RCC (Reuter, 2006). Even for the localized ccRCC, the tumor recurrence and progression can also be observed in ~25% of patients after primary treatment (De et al., 2014). Therefore, there is still an urgent need to explore effective prognostic markers and fully clarify the molecular mechanism underlying the tumorigenesis of ccRCC.

Currently, the risk stratification and prognostic prediction for the ccRCC patients are mainly conducted using the Fuhrman grade and tumor–node–metastasis stage system. Even patients with similar clinicopathologic characteristics could also suffer from variable survival outcomes. Taking into account the heterogeneity in ccRCC, several potential biomarkers for diagnosis and survival prediction have been reported in recent years, such as carbonic anhydrase 9 (Genega et al., 2010), PBRM1, and BAP1 mutation (Varela et al., 2011). However, few of these potential biomarkers could finally be transformed into clinical practice due to the unsatisfactory accuracy and sensibility.

The modification and epigenetic alteration of RNA have recently been primarily identified, including N6-methyladenosine (m^6^A) (Roundtree et al., 2017; Boccaletto et al., 2018). M^6^A RNA methylation acts as one kind of conserved internal modifications in eukaryotic nuclear RNAs (Dubin and Taylor, 1975), which is one of the most common RNA modifications and plays diverse roles in various biological processes (Bokar et al., 1997; Dominissini et al., 2012). The m^6^A status in a cell is regulated by three types of m^6^A RNA methylation genes, including methyltransferases called writers (RBM15, METTL14, KIAA1429, and others), readers (FMR1, IGF2BP1, YTHDC1, and others), and demethylases called erasers (ALKBH5 and FTO) (Li A. et al., 2017). At present, the low expression levels of FTO and ALKBH5 have been proved to be associated with worse cancer-specific survival of RCC patients after nephrectomy (Strick et al., 2020). However, the specific role of m^6^A regulators in carcinogenesis and progression of ccRCC has not been fully understood by now.

In this study, we carried out a comprehensive analysis based on the expressions of m^6^A RNA methylation regulator to identify their important roles in the diagnosis and survival prediction for the ccRCC patients.

## Materials and Methods

### Datasets Source and Screen

Data sets from The Cancer Genome Atlas (TCGA) and Gene Expression Omnibus (GEO) were strictly screened for this study. The inclusion criteria were as follows: studies with more than 60 ccRCC patients and studies with complete clinical data and gene expression data of m^6^A RNA methylation genes. Five GEO datasets, including GSE46699 (Eckel-Passow et al., 2014), GSE53757 (von Roemeling et al., 2014), GSE22541 (Wuttig et al., 2012), GSE17895 (Dalgliesh et al., 2010), and GSE40435 (Wozniak et al., 2013), were finally selected and downloaded with gene expression data and clinical data. Four datasets (GSE46699, GSE53757, GSE22541, and GSE17895) with microarray data were detected based on the Affymetrix Human Genome U133 Plus 2.0 Array. We firstly performed background adjustment and quantile normalization from the raw CEL data of the four datasets by the Robust Multi-array Average in the R environment (Irizarry et al., 2003). And then, we annotated the probe of each gene by using the hgu133plus2.db in R. Finally, the processed microarray data from the four datasets were merged as the Meta-cohort after batch normalization. The processed expression data of GSE40435 based on the Illumina HumanHT-12 V4.0 was directly downloaded from the GEO database, as it had been normalized in a previous study (Wozniak et al., 2013), which was defined as the GSE-cohort. TCGA-KIRC data set, including normalized RNA-sequencing data and clinical data, was directly downloaded from the TCGA database. Expression data files of 21 m^6^A RNA methylation genes were further extracted from the processed data sets mentioned earlier. Basic clinical characteristics of data sets in this study are shown in Supplementary Table 1.

### Construction of the Diagnostic and Prognosis Model Based on the m^6^A Regulators for Clear-Cell Renal Cell Carcinoma

Differential expression analysis of the 21 m^6^A RNA methylation genes between normal and tumor samples was performed in the Meta-cohort and the GSE-cohort. Only m^6^A RNA methylation genes differentially expressing in both the two cohorts were further used for the construction of the diagnostic model. We carried out the least absolute shrinkage and selection operator (LASSO) analysis via the *glmnet* package to identify ccRCC-related m^6^A regulators and calculate coefficients of each gene in the Meta-cohort. The number of lambda values in LASSO was set as 1,000 to ensure the robustness of our diagnostic models. The m^6^A diagnostic score was then calculated as follow:

$$\begin{array}{l} \text{m}6\text{A diagnostic score }=\sum_{i\;=\;1}^{n}\left(\text{Coefi}*\text{Di}\right) \end{array}$$

Coefi means the coefficient of each ccRCC-related m^6^A RNA methylation gene, whereas Di represents the related messenger RNA (mRNA) expression. The diagnostic model was further verified in two independent cohorts (GSE-cohort and TCGA-cohort).

To construct the prognosis model based on the m^6^A regulators for ccRCC, 531 ccRCC patients from the TCGA cohort were randomly assigned to a training cohort and a testing cohort by the random number method. We performed a LASSO Cox regression analysis to identified survival-related m^6^A RNA methylation genes and calculated their coefficients in the TCGA training cohort. The m^6^A risk score was then calculated as follow:

$$\begin{array}{l} \text{m}6\text{A risk score}=\sum_{i\;=\;1}^{n}\left(\text{Coefi}*\text{Ri}\right) \end{array}$$

Coefi means the coefficient of each survival-related m^6^A RNA methylation gene, whereas Ri represents the related mRNA expression. The prognosis model was further validated in the TCGA testing cohort and the independent validation cohort (GSE22541). The cutoff value of high and low m^6^A risk score was defined as the median score of respective cohorts.

### Development and Evaluation of the Prognostic Nomogram Model

We developed a prognostic nomogram model based on m^6^A risk score, age, tumor stage, and tumor grade using the *rms* and *nomogramEx* packages in the R environment. The calibration with bootstrapping was conducted to verify the nomogram-predicted probabilities of the 1-, 3-, and 5-year overall survival (OS) via plotting on the x-axis, with actual OS probabilities plotting on the y-axis. The time-dependent receiver operating characteristic (ROC) curve analysis and the decision curve analysis were also performed to identify the specificity, sensitivity, and clinical utility of the prognostic model.

### Weighted Gene Co-expression Network and Functional Enrichment Analysis

The significantly differentially expressed genes (DEGs) were firstly identified between the ccRCC tissue and the normal renal tissue from GSE40435, GSE53757, and GSE46699. The weighted gene co-expression network analysis (WGCNA) was then performed based on the DEGs by the *WGCNA* package in R (Langfelder and Horvath, 2008). When the soft-thresholding power of β value was defined as 8, the recruited DEGs were hierarchically clustered into seven gene modules. The correlation between gene module and clinical characteristic was further analyzed to identify the optimal gene module with the highest correlation. We finally conducted the Kyoto Encyclopedia of Genes and Genomes pathway and the Genetic Ontology analysis by the Metascape (Zhou et al., 2019) to explore the potential biological mechanisms in which the m^6^A risk score might be involved in.

### Statistical Analysis

The continuous variable with abnormal distribution was analyzed by non-parametric tests (Mann–Whitney *U* test for comparison between two groups and Kruskal–Wallis test for comparisons among more than two groups). The log-rank test was used for analyzing the Kaplan–Meier curves of OS and disease-free survival. The Cox regression analyses were carried out to identify the m^6^A risk score as a prognostic factor of OS. R (3.6.2) and Statistical Package for Social Sciences 24.0 software (SPSS Inc., Chicago, IL, USA) were used for statistical analysis and data visualization. A two-tailed *P*-value of <0.05 was considered significant.

## Results

### Important Roles of m^6^A Regulators in Clear-Cell Renal Cell Carcinoma

**Figure 1:**
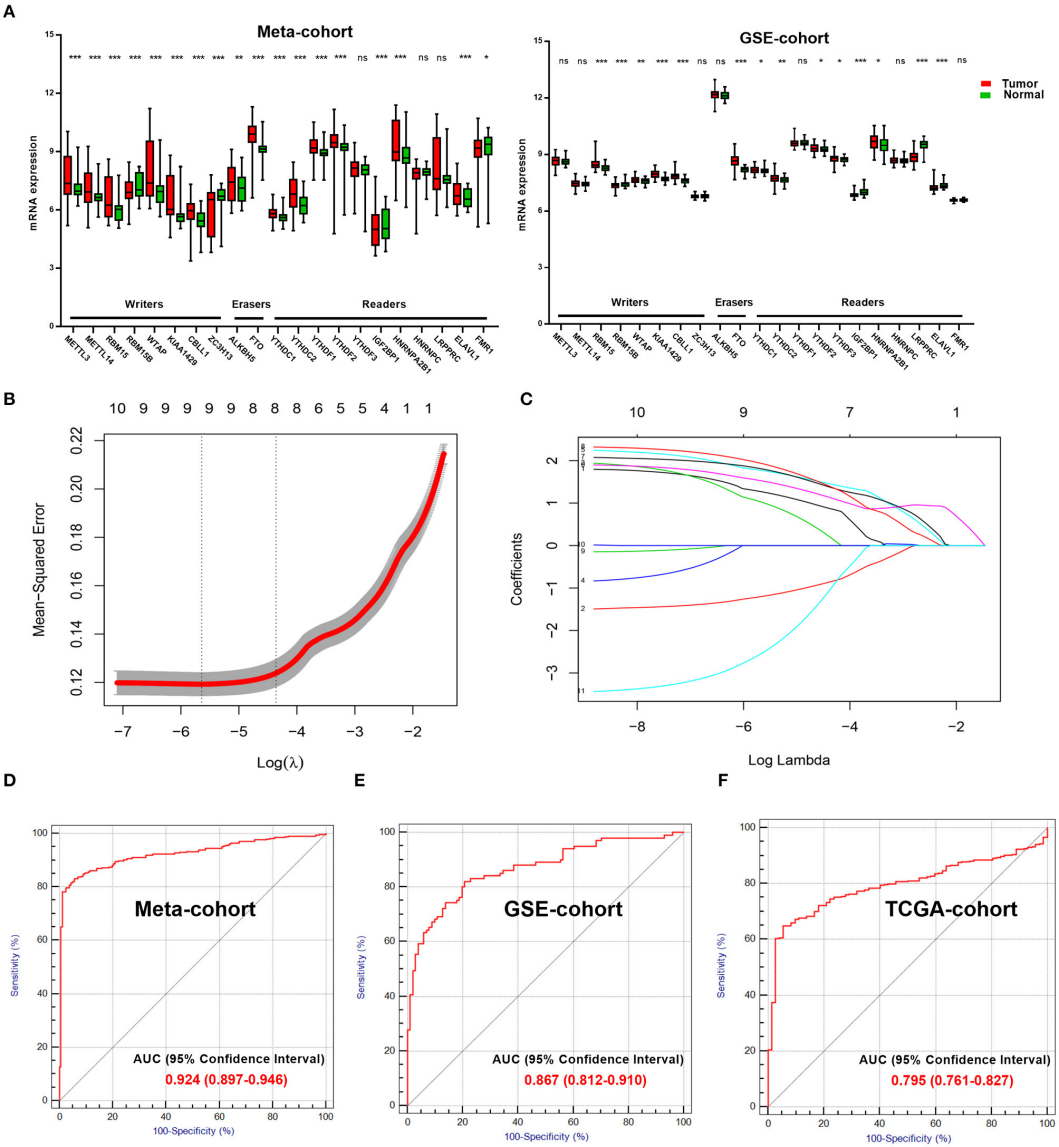
Important role of m^6^A regulators in ccRCC. **(A)** Exploring (Meta-cohort) and verifying (GSE-cohort) different mRNA expression of 21 m^6^A regulators between normal and tumor samples in ccRCC. **(B)** 10-fold cross-validated error for LASSO analysis. First vertical line equals minimum error, whereas second vertical line shows cross-validated error within one standard error of minimum. **(C)** Profile of coefficients in model at varying levels of penalization plotted against log (lambda) sequence. **(D)** ROC curve of diagnostic score in training cohort. **(E,F)** ROC curves of diagnostic score in validation cohorts (GSE-cohort and TCGA-cohort). m^6^A, N^6^-methyladenosine; ccRCC, clear-cell renal cell carcinoma; LASSO, least absolute shrinkage and selection operator; ROC, receiver operating characteristic; TCGA, The Cancer Genome Atlas.

We explored and verified 21 m^6^A regulators with differential mRNA expressions between the tumor samples and normal samples in the Meta-cohort and the GSE-cohort (Figure 1A). Only 11 m^6^A regulators were significantly differentially expressed in both the two cohorts, including five writers (RBM15, RBM15B, WTAP, KIAA1429, and CBLL1), one eraser (FTO), and five readers (YTHDC1, YTHDC2, YTHDF2, IGF2BP1, and HNRNPA2B1). The 11 m^6^A regulators were further analyzed by the LASSO analysis in the Meta-cohort (Figure 1B). Finally, eight ccRCC-related m^6^A RNA methylation genes, including RBM15, WTAP, CBLL1, FTO, YTHDC1, YTHDC2, YTHDF2, and HNRNPA2B1, were selected for the construction of the m^6^A-related diagnostic model (Figure 1C). The ROC analysis revealed that the area under the curve (AUC) of the diagnostic model reached 0.924 [95% confidence interval (CI): 0.897–0.946] in Meta-cohort (Figure 1D). This diagnostic model was further validated in two independent cohorts, including GSE-cohort and TCGA-cohort. AUC values reached 0.867 (95% CI: 0.812–0.910) in GSE-cohort (Figure 1E) and 0.795 (95% CI: 0.761–0.827) in TCGA-cohort (Figure 1F), which proved the stability of the diagnostic efficiency of our m^6^A-related diagnostic model for the ccRCC patients.

### Effective Survival Prediction of Clear-Cell Renal Cell Carcinoma Patients by the m^6^A Risk Score

**Figure 2:**
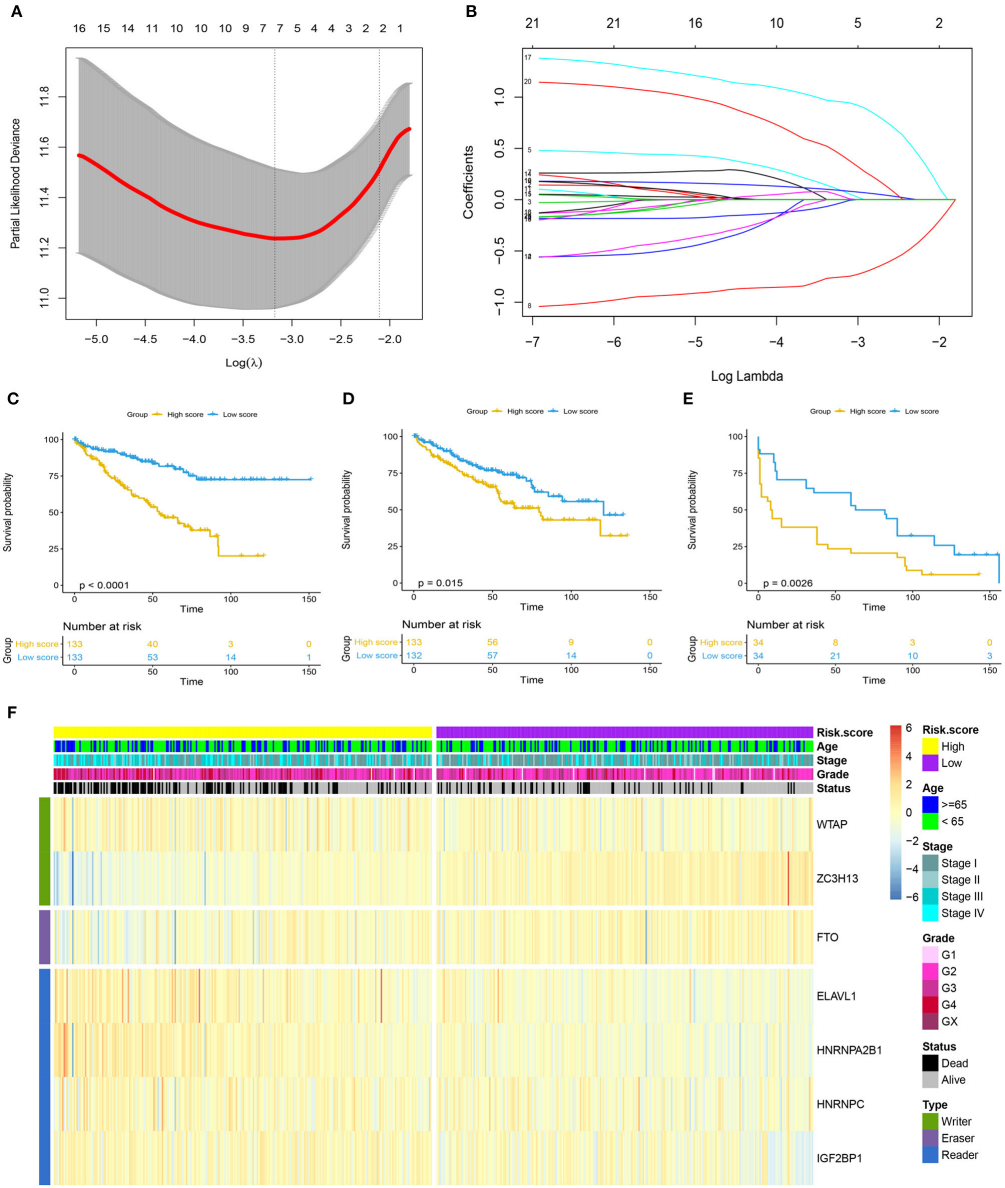
Prognosis model based on m^6^A regulators for ccRCC. **(A)** 10-fold cross-validated error for LASSO analysis. First vertical line equals minimum error, whereas second vertical line shows cross-validated error within one standard error of minimum. **(B)** Profile of coefficients in model at varying levels of penalization plotted against log (lambda) sequence. **(C–E)** Kaplan–Meier survival analysis of overall survival stratified by m^6^A risk score for ccRCC patients in TCGA training cohort, TCGA validation cohort, and disease-free survival in independent validation cohort (GSE22541). **(F)** Heatmap summarized expression of seven m^6^A methylation genes selected by LASSO Cox regression analysis and distribution of clinicopathologic factors in whole TCGA cohort. Cutoff value of high and low m^6^A risk score was defined as median score of respective cohorts. m^6^A, N^6^-methyladenosine; ccRCC, clear-cell renal cell carcinoma; LASSO, least absolute shrinkage and selection operator; TCGA, The Cancer Genome Atlas.

As shown in Figure 2A, the first vertical line pointed at 7 in the TCGA training cohort, which equaled the minimum 10-fold cross-validated error, indicating that seven optional prognosis-related m^6^A regulators, including WTAP, ZC7H13, FTO, ELAVL1, HNRNPA2B1, HNRNPC, and IGF2BP1, were selected by the LASSO Cox regression analysis (Figure 2B). A significant difference was observed in the OS [hazard ratio (HR) = 3.474, 95% CI: 2.260–5.342, *p* < 0.0001] between the ccRCC patients with high and low m^6^A risk score in the TCGA training cohort (Figure 2C), which was further verified in the TCGA testing cohort (Figure 2D) with HR value of 1.679 (95% CI: 1.113–2.532, *p* = 0.015) for the OS and HR value of 2.101 (95% CI: 1.186–3.724, *p* = 0.001) for the disease-free survival in the independent GSE22541 cohort (Figure 2E). A higher m^6^A risk score was correlated with the higher tumor stage, higher tumor grade, and death (Figure 2F).

**Figure 3:**
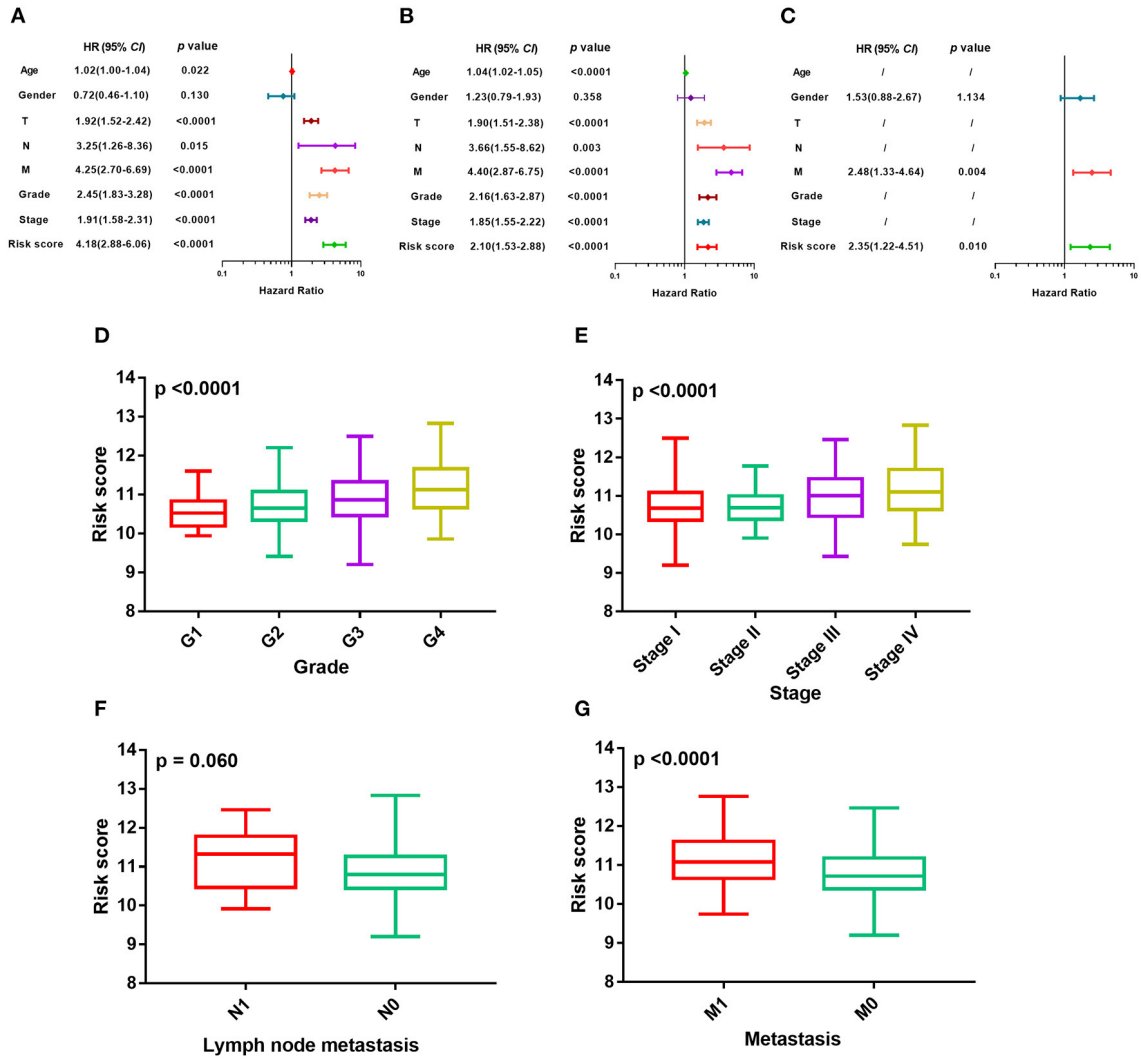
Evaluation of prognosis model based on m^6^A regulators. **(A–C)** Univariate Cox regression analyses of m^6^A risk score and clinicopathologic factors in TCGA training cohort, TCGA validation cohort, and independent validation cohort. **(D–G)** Difference in distribution of m^6^A risk score of different tumor stages, tumor grades, metastasis status, and lymph node metastasis status in whole TCGA cohort. m^6^A, N^6^-methyladenosine; TCGA, The Cancer Genome Atlas.

The m^6^A risk score was identified as a risk factor for the OS in ccRCC patients by the univariate Cox regression analysis (Figure 3A), which was further verified in both the TCGA training cohort (Figure 3B) and the independent GSE22541 cohort (Figure 3C). There existed significant differences of the m^6^A risk score among different tumor grades (*P* < 0.0001, Figure 3D), different tumor stages (*P* < 0.0001, Figure 3E), different tumor lymph node metastasis status (*P* = 0.06, Figure 3F), and different tumor distant metastasis status (*P* < 0.0001, Figure 3G).

### Prognostic Accuracy of the m^6^A Risk Score Integrated With the Clinicopathologic Factors

**Figure 4:**
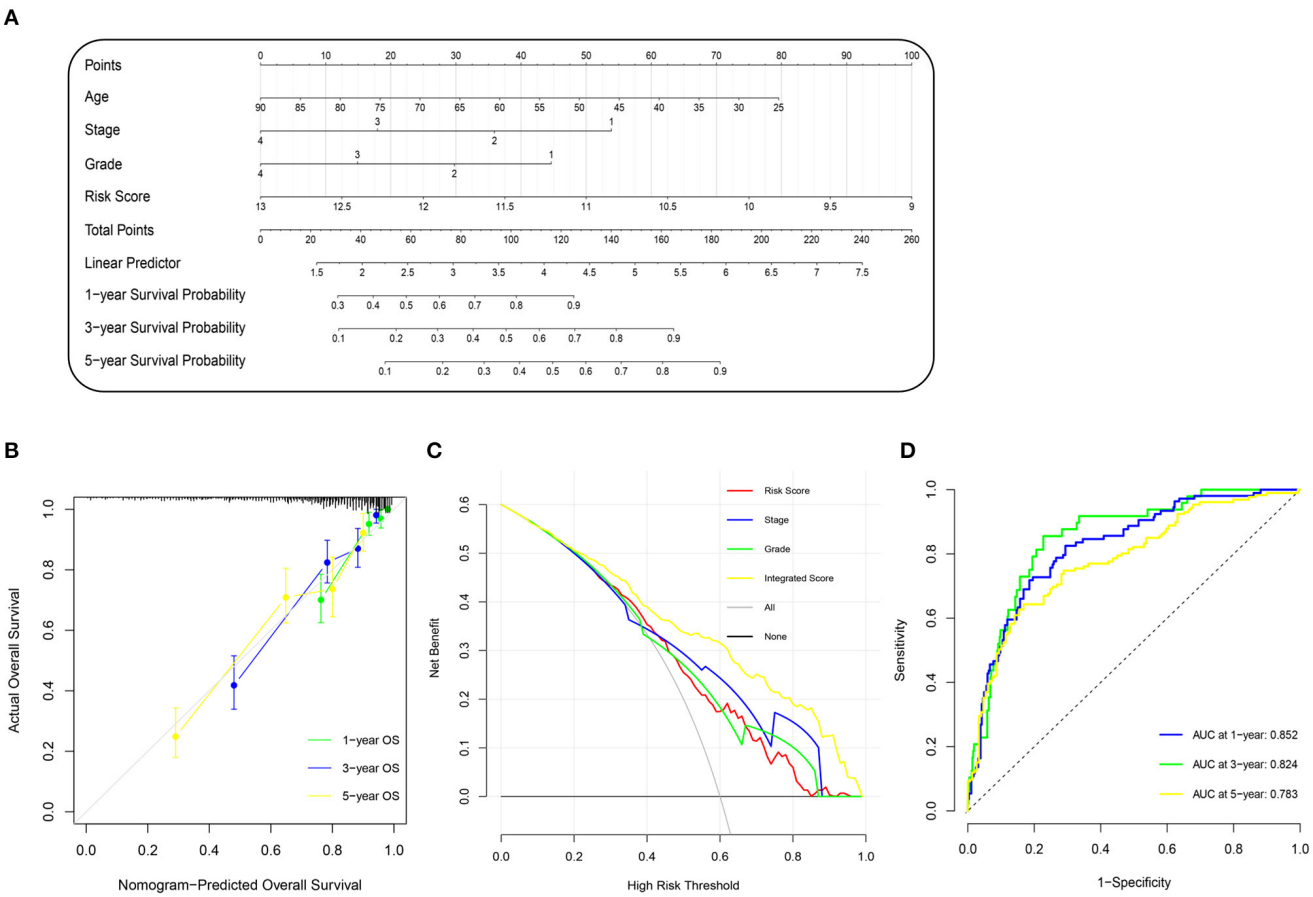
Construction and evaluation of nomogram prognosis model in TCGA cohort. **(A)** Nomogram based on m^6^A risk score and clinicopathologic factors for 1-, 3-, and 5-year overall survival prediction of ccRCC patients. **(B)** Evolution of prognostic nomogram model for 1-, 3-, and 5-year overall survival prediction. **(C)** Decision curve analysis compared overall survival benefits among nomogram, tumor stage, tumor grade, and m^6^A risk score. Black line: all victims dead. Gray line: none victims dead. **(D)** Time-dependent ROC curve of 1-, 3-, and 5-year overall survival prediction for prognostic nomogram model. m^6^A, N^6^-methyladenosine; TCGA, The Cancer Genome Atlas; ccRCC, clear-cell renal cell carcinoma; ROC, receiver operating characteristic.

To improve the accuracy of our survival prognostic model, we developed an integrated nomogram by combining the m^6^A risk score and predictable clinicopathologic factors, including age, tumor grade, and tumor stage. The integration nomograms for the OS prediction are shown in Figure 4A. The calibration plots revealed that the 1-, 3-, and 5-year OS probabilities predicted by the integrated nomogram model had an excellent agreement with the actual observations (Figure 4B), indicating good performance in predicting the survival status of the ccRCC patients. The decision curve analysis illustrated that when the threshold probability was more than 0.2, the integrated nomogram for the OS prediction could be more favorable than either the m^6^A risk score and the predictable clinicopathologic factors (Figure 4C). Further time-dependent ROC curve revealed that the AUC values of the integration nomogram for the OS prediction in 1, 3, and 5 years arrived at 85.2, 82.4, and 78.3%, respectively (Figure 4D).

### Immune-Related Pathways Were Associated With the m^6^A RNA Methylation Risk Signature

**Figure 5:**
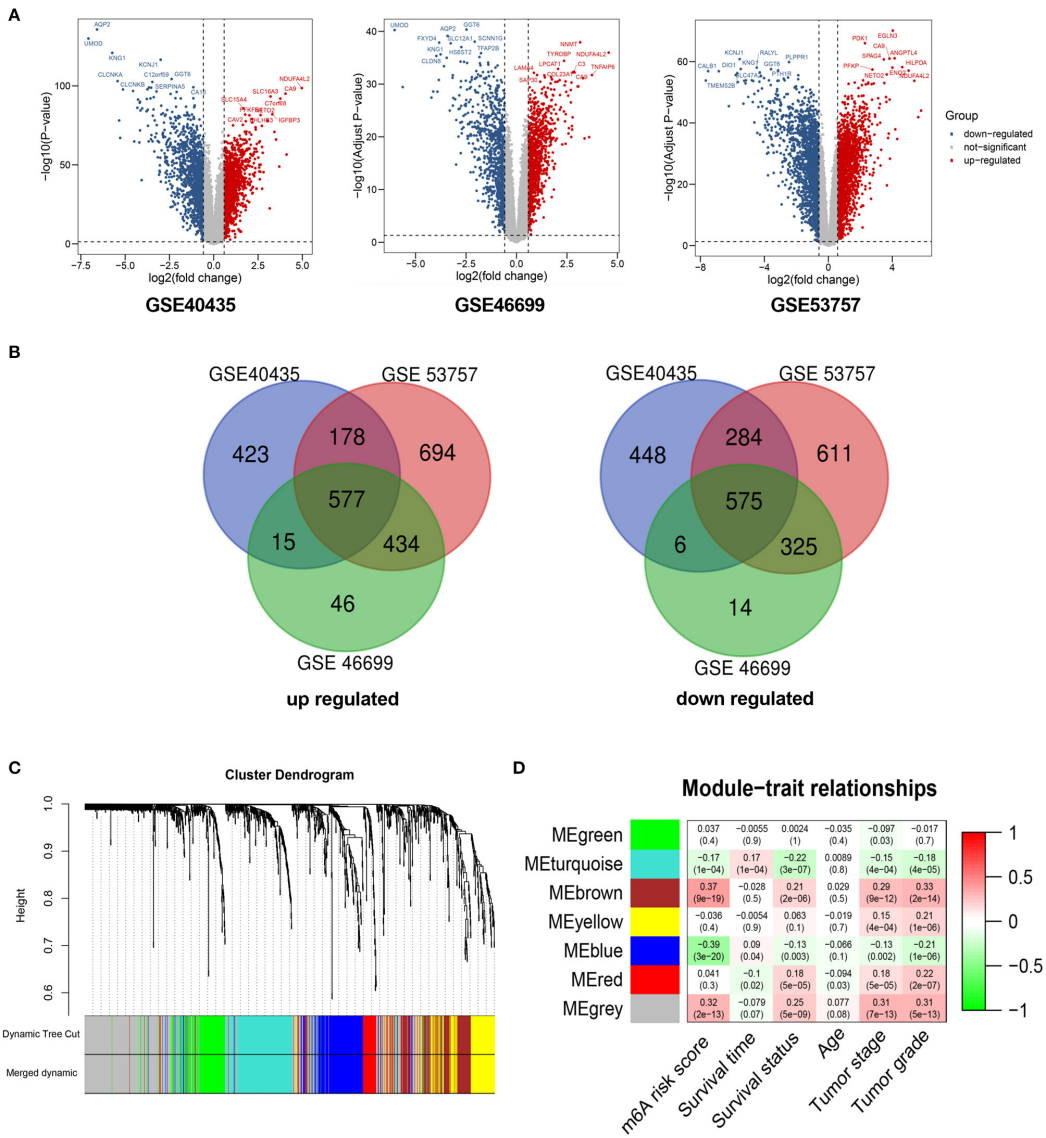
WGCNA for differentially expressed genes in ccRCC. **(A)** Volcano plots showing significantly upregulated and downregulated genes in GSE40435, GSE53757, and GSE46699. **(B)** Venn diagrams presenting intersections of significantly upregulated and downregulated genes among GSE40435, GSE53757, and GSE46699. **(C)** Gene dendrogram with different colors showing modules identified by WGCNA. **(D)** Relationship between gene modules and clinical characteristic. WGCNA, weighted gene co-expression network analysis; ccRCC, clear-cell renal cell carcinoma.

**Figure 6:**
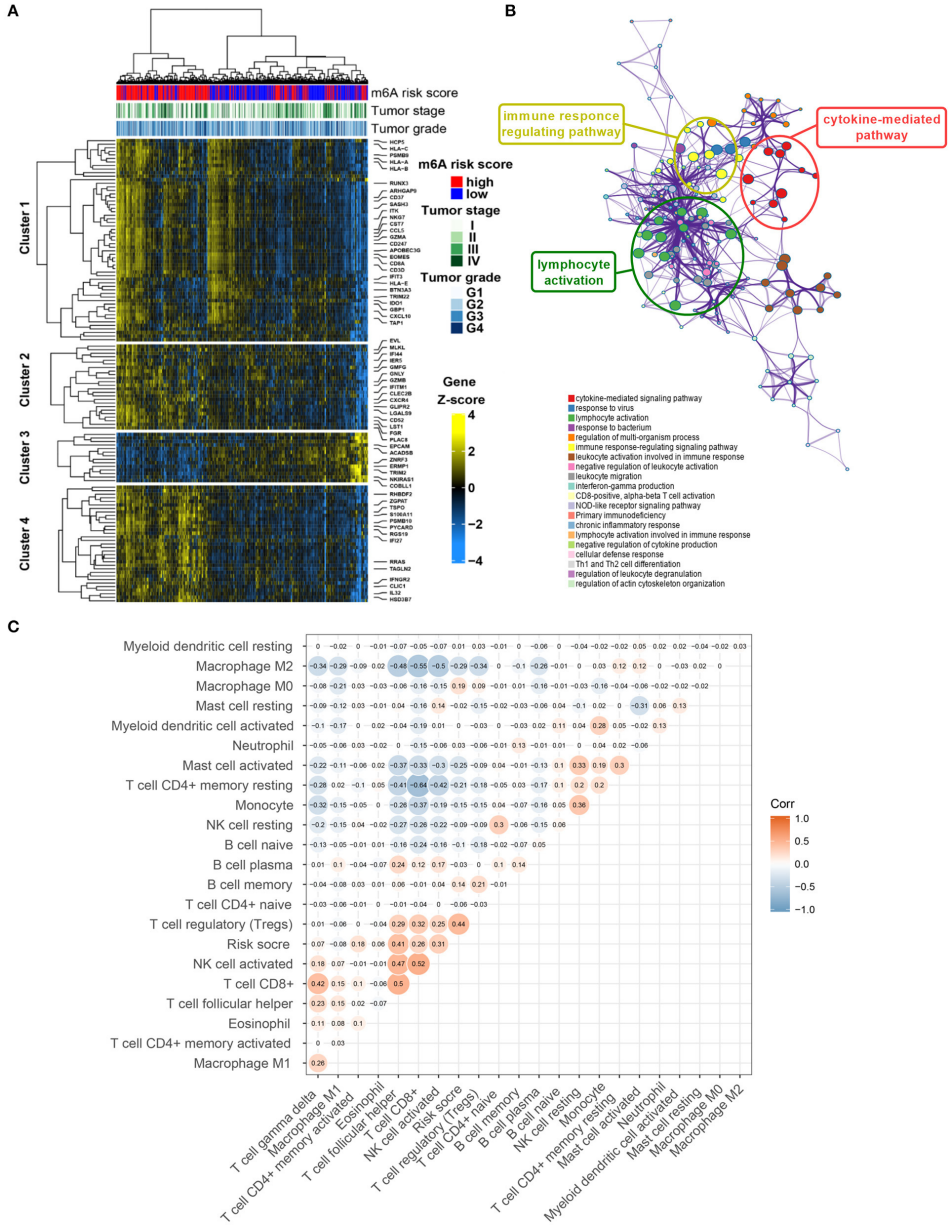
Potential mechanisms of co-expressed genes associated with m^6^A risk score. **(A)** Heatmap visualizing expressions of co-expressed genes in brown module. **(B)** Potentially enriched pathways of co-expressed genes in brown module. **(C)** Correlation analysis of m^6^A risk score and abundance of 22 types of immune cells. m^6^A, N^6^-methyladenosine.

**Figure 7:**
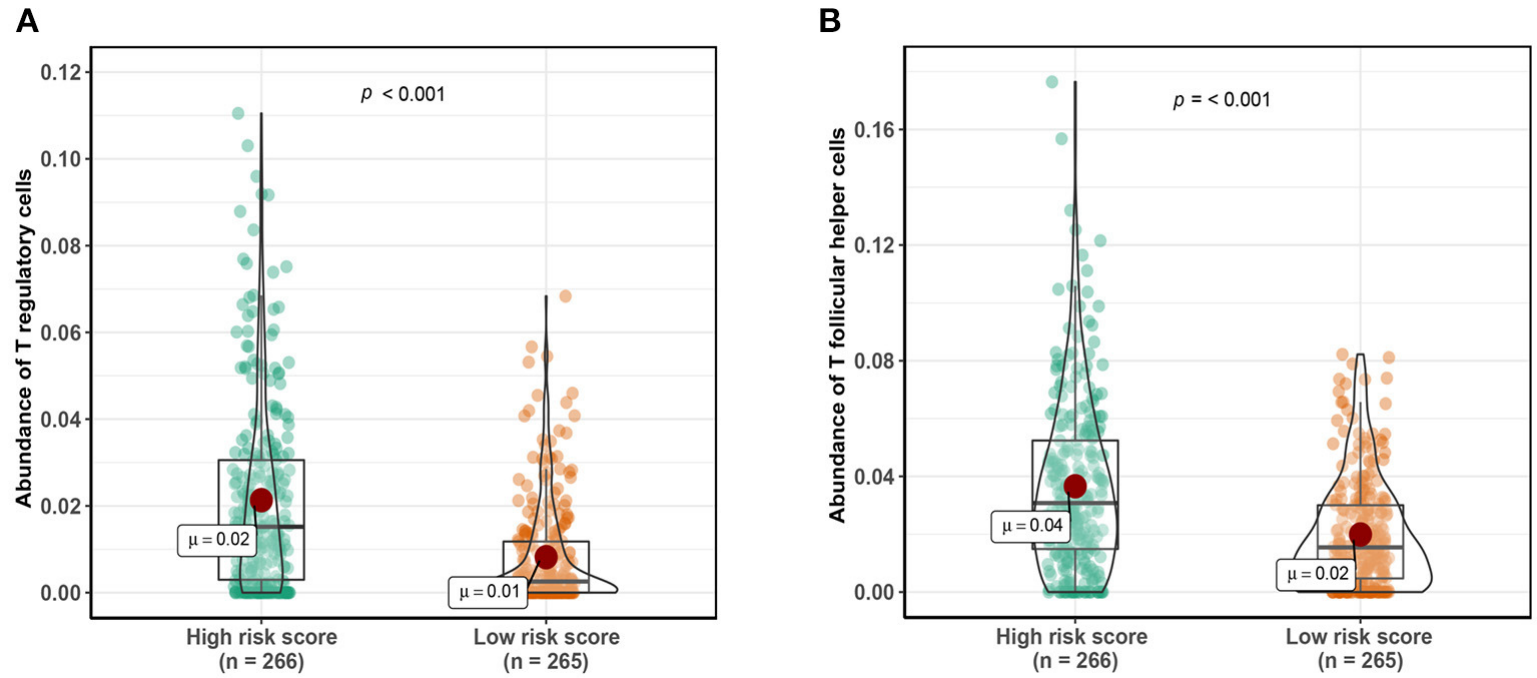
Comparing abundance of T regulatory cells **(A)** and T follicular helper cells **(B)** between patients with high-risk scores and low-risk scores. Cutoff value of high- and low-risk scores was defined as median score.

A total of 1,152 DEGs were identified in the GSE40435 cohort, GSE53757 cohort, and GSE46699 cohort (Figure 5A), including 577 upregulated genes and 570 downregulated genes (Figure 5B). The DEGs were hierarchically clustered into seven gene modules by the WGCNA method (Figure 5C), and the brown model was found to perform the highest correlation to the m^6^A RNA methylation risk signature (Figure 5D). Significantly different expressions of 128 genes in the brown model were observed between the patients with high and low m^6^A risk scores (Figure 6A). As shown in Figure 6B, some immune-related pathways, including the immune response regulating pathway, the cytokine-mediated pathway, and the lymphocyte activation-associated pathway, were enriched in genes from the brown model, suggesting that m^6^A RNA methylation might affect clinical prognosis through regulating immune functions of ccRCC patients. Further correlation analysis revealed that the m^6^A risk score was significantly correlated with the abundances of T regulatory cells (Tregs) and T follicular helper cells (Tfhs) (Figure 6C). Higher abundances of the Tregs and the Tfhs were found in the patients with high m^6^A risk scores (Figures 7A,B).

## Discussion

Due to the high incidence and mortality of ccRCC, the accurate diagnosis and survival prediction of ccRCC patients were urgently needed. Thanks to the innovative developments in high-throughput genetic diagnostic techniques for oncology, there has been a revolutionary improvement in the efficient diagnosis of a malignant tumor at the molecular level (Cancer Genome Atlas Research Network, 2013; Linehan et al., 2016; Zehir et al., 2017).

In this study, we designed a diagnostic model for ccRCC based on the m^6^A regulators. The ccRCC and normal renal tissue could be accurately told out by the m^6^A diagnostic score in Meta-cohort, and the result was further verified in the independent GSE-cohort and TCGA-cohort, with the AUC values of 0.924, 0.867, and 0.795, respectively. Effective survival prediction of ccRCC by the m^6^A risk score was also identified in the TCGA training cohort and validated in the testing cohort and the independent GSE22541 cohort, with the HR values of 3.474, 1.679, and 2.101 for clinical survival, respectively. It was proved that the m^6^A risk score in ccRCC was associated with higher tumor stage, higher tumor grade, and tumor metastasis. All of these results suggested the crucial roles of m^6^A regulators in regulating tumorigenesis and tumor progression, as previously reported (He et al., 2019; Wang et al., 2019; Tian et al., 2020).

An integrated nomogram by combining m^6^A risk score and predictable clinicopathologic factors, including age, tumor grade, and tumor stage, was constructed in this study to improve the accuracy of our survival prognostic model. The time-dependent ROC curve revealed that the AUC values of the integration nomogram for OS prediction in 1, 3, and 5 years arrived at 85.2, 82.4, and 78.3%, respectively, indicating advantageous usability of our survival prediction model in clinical practice.

Different types of m^6^A RNA methylation regulators work differently in tumorigenesis. For example, FTO could promote the progression of lung carcinoma by releasing the m^6^A modification in MZF1 mRNA and strengthening its stability (Liu et al., 2018). However, METTL14 was found to suppress RCC by downregulating P2RX6 protein translation (Gong et al., 2019). Generally, writers could irritate m^6^A modifications in the mRNA of tumor suppressor genes or oncogenes. And then, these modifications could be recognized by readers, resulting in downregulating tumor suppressor or upregulating oncogene expression (Wang et al., 2020).

A total of 128 DEGs associated with the m^6^A RNA methylation risk signature in the brown model were enriched in immune regulated pathways, including the immune response regulating pathway, the cytokine-mediated pathway, and the lymphocyte activation-related pathway. In addition, the m6A risk score was significantly correlated with the abundance of Tregs and Tfhs. The important role of m^6^A modification in immune cell-related pathogenesis through the m^6^A mediated degradation of Naïve T cell had been recently identified (Li H. B. et al., 2017). Our enrichment results indicated that the m^6^A RNA methylation in ccRCC might affect the prognosis through regulating immune function, especially the functions of Tregs and Tfhs.

There are also several defects in this study. Firstly, our main findings were based on integrated bioinformatics analyses, without experimental verification. The experiment verification at the cellular level, including regulation mechanism research and function verification, are still needed. Secondly, all of the data sets analyzed in this study were acquired from the public database, resulting in a potential bias in genetic and clinical data. However, cross-validation among independent datasets has been performed as much as possible to reduce the potential bias. Thirdly, the cutoff value of the high and low m^6^A risk score group was defined as the median score of respective cohorts. Actually, the optional cutoff value of m^6^A risk score to distinguish the patients with high survival risk is supposed to be identified and verified in larger clinical patient cohorts. Finally, our work still requires further clinical validation to verify its application to the clinic in ccRCC patients.

## Conclusions

In summary, we identified the important role of the m^6^A regulator in ccRCC. Immune-related pathways might be involved in the regulation of ccRCC through the m^6^A RNA methylation. The novel m^6^A gene signature might act as an effective biomarker for prognosis prediction of the ccRCC patients, which still requires further clinical validation and experimental verification.

## Data Availability Statement

The original contributions generated for the study are included in the article/Supplementary Material, further inquiries can be directed to the corresponding authors.

## Author Contributions

JZ, XW, and SC conceived the study, designed the research, and wrote the paper. SC, EZ, NZ, and TW conducted and analyzed experiments. LJ and XW provided samples. JZ supervised the research. All authors contributed to the article and approved the submitted version.

## Conflict of Interest

The authors declare that the research was conducted in the absence of any commercial or financial relationships that could be construed as a potential conflict of interest.
