# Supplementary material for: A Novel m6A Gene Signature Associated With Regulatory Immune Function for Prognosis Prediction in Clear-Cell Renal Cell Carcinoma
Source: Front Cell Dev Biol. 2021 Jan 21;8:616972. doi: 10.3389/fcell.2020.616972 (PMC7858250; doi:10.3389/fcell.2020.616972)
Supplement: Supplementary file 1 [file Table_1.DOCX]

**TABEL S1** Basic clinical characteristics of datasets in this study.

| **Dataset** | **Platform** | **No. of normal samples** | **No. of tumor samples** | **Tumor stage** | **Tumor grade** | **Gender** | **Age** | **Region** | **Survival outcome** |
| --- | --- | --- | --- | --- | --- | --- | --- | --- | --- |
| **GSE46699** | Affymetrix Human Genome | 65 | 65 | pT1: 46 | G1: 10 | Male: 34 | <50: 9 | USA | NA |
|  | U133 Plus 2.0 Array |  |  | pT2: 6 | G2: 37 | Female: 31 | ≥50: 55 |  |  |
|  |  |  |  | pT3: 13 | G3: 18 |  | Unknown: 1 |  |  |
| **GSE53757** | Affymetrix Human Genome | 72 | 72 | Stage I: 24 | NA | NA | NA | USA | NA |
|  | U133 Plus 2.0 Array |  |  | Stage II: 19 |  |  |  |  |  |
|  |  |  |  | Stage III: 14 |  |  |  |  |  |
|  |  |  |  | Stage IV: 15 |  |  |  |  |  |
| **GSE22541** | Affymetrix Human Genome | 0 | 68 | pT1: 10 | G2: 18 | Male: 44 | NA | Germany | Disease-free survival |
|  | U133 Plus 2.0 Array |  |  | pT2: 9 | G3: 6 | Female: 24 |  |  |  |
|  |  |  |  | pT3: 5 | Unknown: 44 |  |  |  |  |
|  |  |  |  | Unknown: 44 |  |  |  |  |  |
| **GSE17895** | Affymetrix Human Genome | 22 | 138 | Stage I: 44 | G1: 4 | Male: 56 | NA | USA | NA |
|  | U133 Plus 2.0 Array |  |  | Stage II: 14 | G2: 35 | Female: 40 |  |  |  |
|  |  |  |  | Stage III: 34 | G3: 39 | Unknown: 32 | |  |  |
|  |  |  |  | Stage IV: 2 | G4: 16 |  |  |  |  |
|  |  |  |  | Unknown: 44 | Unknown: 44 |  |  |  |  |
| **GSE40435** | Illumina HumanHT-12 V4.0 | 101 | 101 | Stage I: 28 | G1: 14 | Male: 59 | <55: 20 | France | NA |
|  |  |  |  | Stage II: 6 | G2: 27 | Female: 42 | ≥55: 81 |  |  |
|  |  |  |  | Stage III: 7 | G3: 13 |  |  |  |  |
|  |  |  |  | Stage IV: 5 | G4: 5 |  |  |  |  |
|  |  |  |  | Unknown: 13 |  |  |  |  |  |
| **TCGA** | Illumina RNAseq | 72 | 531 | Stage I: 266 | G1: 13 | Male: 345 | <55: 173 | NA | Overall survival |
|  |  |  |  | Stage II: 57 | G2: 229 | Female: 186 | ≥55: 358 |  |  |
|  |  |  |  | Stage III: 124 | G3: 205 |  |  |  |  |
|  |  |  |  | Stage IV: 84 | G4: 76 |  |  |  |  |
|  |  |  |  |  | Unknown: 8 |  |  |  |  |

NA: not appliable.
